# Clinical and Lifestyle-Related Prognostic Indicators among Esophageal Adenocarcinoma Patients Receiving Treatment at a Comprehensive Cancer Center

**DOI:** 10.3390/cancers13184653

**Published:** 2021-09-16

**Authors:** Shruti G. Dighe, Li Yan, Sarbajit Mukherjee, Cailey S. McGillicuddy, Karen L. Hulme, Steven N. Hochwald, Saikrishna Yendamuri, Andrew J. Bain, Kevin T. Robillard, Kirsten B. Moysich, Christine B. Ambrosone, Amy E. Millen, Matthew F. Buas

**Affiliations:** 1 Department of Cancer Prevention and Control, Roswell Park Comprehensive Cancer Center, Buffalo, NY 14263, USA; cailey.mcgillicuddy@roswellpark.org (C.S.M.); karen.hulme@roswellpark.org (K.L.H.); moysich@roswellpark.org (K.B.M.); christine.ambrosone@roswellpark.org (C.B.A.); 2 Department of Biostatistics and Bioinformatics, Roswell Park Comprehensive Cancer Center, Buffalo, NY 14263, USA; li.yan@roswellpark.org; 3 Department of Medicine, Roswell Park Comprehensive Cancer Center, Buffalo, NY 14263, USA; sarbajit.mukherjee@roswellpark.org (S.M.); andrew.bain@roswellpark.org (A.J.B.); Kevin.Robillard@roswellpark.org (K.T.R.); 4 Department of Surgical Oncology, Roswell Park Comprehensive Cancer Center, Buffalo, NY 14263, USA; steven.hochwald@roswellpark.org; 5 Department of Thoracic Surgery, Roswell Park Comprehensive Cancer Center, Buffalo, NY 14263, USA; sai.yendamuri@roswellpark.org; 6 Department of Epidemiology and Environmental Health, School of Public Health and Health Professions, University at Buffalo, State University of New York, Buffalo, NY 14214, USA; aemillen@buffalo.edu

**Keywords:** esophageal adenocarcinoma, survival, prognosis, lifestyle factors

## Abstract

**Simple Summary:**

Esophageal adenocarcinoma (EAC) is a highly lethal cancer with rising incidence in Western countries. Despite diagnostic and therapeutic advances, average 5-year EAC survival remains poor (~20%), with tumor stage and treatment the strongest prognostic factors. The role of lifestyle-related exposures remains uncertain. To address this gap, we analyzed survival associations among EAC patients treated at a tertiary cancer center. Importantly, this study is among the first to assess survival relationships by disease stage for several key lifestyle-related exposures (e.g., physical activity, medications, and diet), enabling us to identify associations which may have been obscured in past analyses. Our findings suggest that lifestyle interventions such as smoking cessation, exercise regimens, and use of cholesterol-lowering (statin) or anti-inflammatory (NSAID) medications may represent promising avenues to improve outcomes in this deadly cancer.

**Abstract:**

Purpose: The incidence of esophageal adenocarcinoma (EAC) has risen substantially in recent decades, while the average 5-year survival remains only ~20%. Disease stage and treatment are the strongest prognostic factors. The role of lifestyle factors in relation to survival remains uncertain, with a handful of studies to date investigating associations with obesity, smoking, physical activity, diet, or medications. Methods: This study included patients diagnosed with primary adenocarcinoma of the esophagus, gastroesophageal junction, or cardia (N = 371) at Roswell Park Comprehensive Cancer Center between 2003 and 2019. Leveraging extensive data abstracted from electronic medical records, epidemiologic questionnaires, and a tumor registry, we analyzed clinical, behavioral, and environmental exposures and evaluated stage-specific associations with survival. Survival distributions were visualized using Kaplan–Meier curves. Cox proportional hazards regression models adjusted for age, sex, stage, treatment, and comorbidities were used to estimate the association between each exposure and all-cause or cancer-specific mortality. Results: Among patients presenting with localized/regional tumors (stages I–III), current smoking was associated with increased overall mortality risk (HR = 2.5 [1.42–4.53], *p* = 0.002), while current physical activity was linked to reduced risk (HR = 0.58 [0.35–0.96], *p* = 0.035). Among patients with stage IV disease, individuals reporting pre-diagnostic use of statins (HR = 0.62 [0.42–0.92], *p* = 0.018) or NSAIDs (HR = 0.61 [0.42–0.91], *p* = 0.016) had improved overall survival. Exploratory analyses suggested that high pre-diagnostic dietary consumption of broccoli, carrots, and fiber correlated with prolonged overall survival in patients with localized/regional disease. Conclusion: Our data suggest that lifestyle exposures may be differentially associated with EAC survival based on disease stage. Future investigation of larger, diverse patient cohorts is essential to validate these findings. Our results may help inform the development of lifestyle-based interventions to improve EAC prognosis and quality of life.

## 1. Introduction

Esophageal cancer, a relatively rare yet lethal malignancy, has a dismal average 5-year survival rate of only ~20% [1]. Esophageal adenocarcinoma (EAC), the predominant histological subtype in Western countries, has risen in incidence significantly in recent decades [2,3,4]. Gastroesophageal reflux (GERD), central obesity, smoking, male sex, and inherited genetics are established risk factors for EAC and its precursor, Barrett’s esophagus (BE) [5,6,7,8,9]. Mortality is higher among older men [10], and among Blacks as compared to Whites [11]. Disease stage and treatment are the strongest known prognostic factors [12,13]. Neoadjuvant therapy prior to surgery has become the standard-of-care for resectable tumors, providing a significant survival advantage relative to surgery alone [14,15]. The role of lifestyle factors in relation to survival remains less certain, with only a handful of studies reported to date [12,16,17,18,19].

Epidemiologic evidence has linked risk of EAC to obesity, independent of GERD [20,21]. However, associations between body mass index (BMI) and EAC survival are conflicting, with some reports suggesting positive relationships [18,19,22], and others no association [12,23]. Most studies have also indicated no survival associations with reflux [12,18,19], apart from a recent Scandinavian analysis reporting improved survival among symptomatic GERD patients [24]. A survival advantage was recently identified for physically active esophageal cancer patients [25], but was not reported in previous EAC-specific analyses [12,18]. Several studies have suggested that smoking and alcohol consumption are not associated with overall survival (OS) or EAC cancer-specific survival (CSS) [12,16,18,19]. By contrast, one recent study reported reduced survival for each 20 pack-year increase in smoking intensity [17], highlighting inconsistencies in current knowledge. Diet, another important modifiable lifestyle factor, has not been intensively investigated in relation to EAC outcomes [26]. With respect to medications, no survival associations have been identified for pre-diagnostic aspirin/NSAID use [12,19], but post-diagnostic use of statins was linked to improved survival [27,28]. The role of pre-diagnostic statins, prescribed commonly among elderly individuals with comorbidities, remains unknown.

To address the above research gaps and re-evaluate inconsistent associations reported across past studies, we investigated the influence of clinical parameters and pre-diagnostic lifestyle factors on EAC survival among >350 patients receiving treatment at Roswell Park Comprehensive Cancer Center. Leveraging extensive data abstracted from electronic medical records, epidemiologic questionnaires, and a tumor registry, we analyzed a broad array of clinical, behavioral, and environmental exposures and further examined stage-specific associations with survival. These analyses may help further inform the development of lifestyle-based interventions to improve EAC prognosis and quality of life.

## 2. Materials and Methods

### 2.1. Study Population

**Figure 1:**
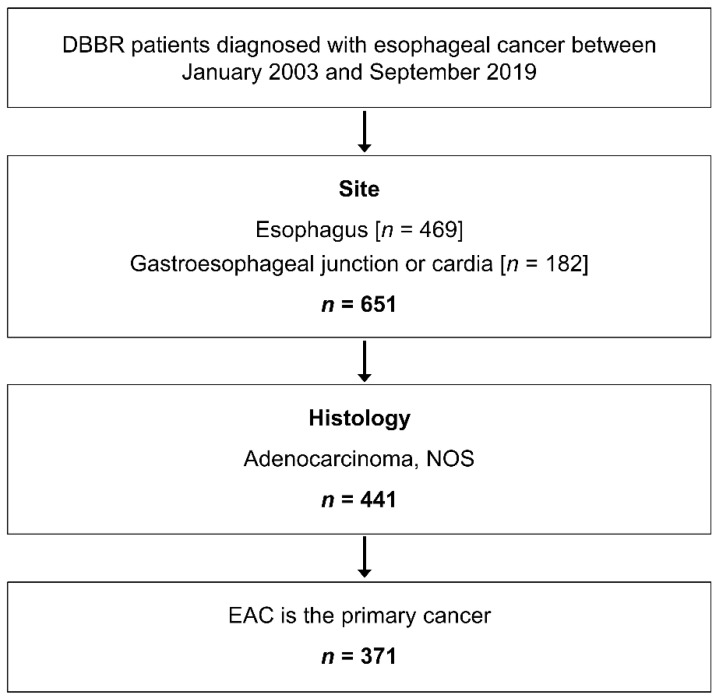
Study population and inclusion criteria.

This study included esophageal cancer (EC) patients with newly diagnosed histologically confirmed primary adenocarcinoma of the esophagus, gastroesophageal junction (GEJ), or cardia (*n* = 371), who consented to participate in the Roswell Park Data Bank and Biorepository (DBBR) between January 2003 and September 2019 (Figure 1). DBBR recruits adult cancer patients diagnosed or treated at Roswell Park Comprehensive Cancer Center. As part of the informed consent, participants complete a self-administered questionnaire, provide a blood sample, and grant permission to link their clinical and biospecimen data.

### 2.2. Data Sources

Baseline and follow-up patient data were obtained from three sources: the electronic medical record (EMR), Roswell Park Tumor Registry, and DBBR questionnaires. The majority of study variables were obtained from the EMR. A structured abstraction instrument was assembled using the secure REDCap application to support abstraction under the Roswell Park Barrett’s and Esophageal Adenocarcinoma Registry (RP-BEAR), a comprehensive database of BE and EAC patients at Roswell Park. Duplicate abstraction was conducted by a second abstractor on ~5% of the study sample to assess reliability. A detailed list of demographic, clinical, and treatment variables is presented in Table S1. Stage at diagnosis was recorded as either stage I, II, III or IV, based on the derived AJCC stage listed under the ‘Tumor Registry’ section of the EMR, or when missing, from the pathological or clinical stage. If all such designations were unavailable, the EMR was reviewed around the time of diagnosis to procure disease stage as mentioned in physician notes. Cases were further classified as ‘localized/regional’ (stages I, II, III) or ‘advanced/metastatic’ (stage IV) [29,30]. Patient outcomes such as follow-up time and last known vital status were collected through the end of September 2019. Vital status was recorded as either ‘alive’ or ‘deceased’. History of comorbidities such as cardiovascular disease, diabetes mellitus, lung, kidney, liver, and cerebrovascular disease were abstracted from medical records (Table S2).

For a subset of participants diagnosed between 2003 and 2016, information pertaining to self-reported physical activity (PA) (*n* = 173) was obtained from the DBBR questionnaires. Approximately 78% of all patients approached consent to enrollment in DBBR. The response rate for questionnaire completion among all consented cancer patients was ~65%. Participants were asked whether they performed any moderate or strenuous exercise in the past 10 years. Responses were categorized as yes, no, or missing. If participants answered yes, they were further questioned regarding the frequency of exercise (days/week, years) and type of exercise, which included moderate/vigorous physical activities (MVPA) such as running, aerobics, swimming, and cycling. To account for current PA, participants reported current involvement in any type of exercise for 20 min or more (around the time of presentation and questionnaire completion). Responses were categorized as yes, no, or missing. BMI at ages 18, 30, 45 and 60 years was calculated from self-reported height at enrollment and self-reported past weight. 

The DBBR questionnaire also included questions pertaining to usual dietary intake. Participants reported their frequency of consumption as servings of foods per month in the year prior to cancer diagnosis. For each frequency category, we assigned a serving number per month (0, <1, 1, 2.5, 4, 8, 14, 21, 30, 45 or missing). Total vegetable and fruit intake variables were computed by summing frequency intake values per month across all individual vegetables and fruits, respectively. Intake of each food item was further categorized into two groups: high intake (corresponding to above median servings) and low intake (corresponding to below median number of servings). Nutrient intake from foods was estimated using the USDA National Nutrient Database standard reference (release 20) [31]. Macronutrients were presented as a percent of total kilocalories (kcal) and further categorized into tertiles. The DBBR questionnaire was adapted from validated questionnaires including the VITamins And Lifestyle cohort study (VITAL) [32,33] and The Diet, Exercise, Lifestyle and Cancer Prognosis Study (DELCaP) [34,35,36].

DBBR and RP-BEAR have been approved by the Roswell Park Institutional Review Board. The DBBR honest broker linked deidentified patient data from all three sources.

### 2.3. Statistical Analysis

**Figure 2:**
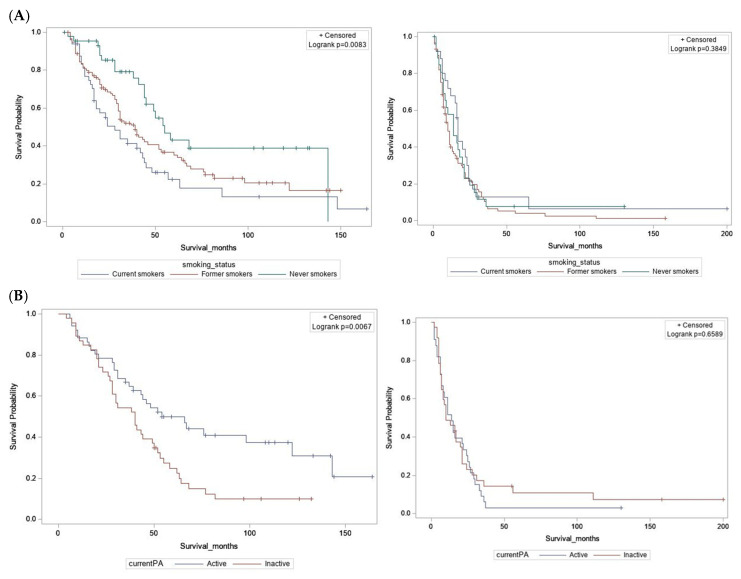
Kaplan–Meier survival curves for overall survival. (**A**) By smoking status at diagnosis. (**B**) By current physical activity. Localized/regional tumors (**left**); advanced/metastatic tumors (**right**).

Differences by vital status were examined using independent sample t-tests for continuous variables, and by Chi-square tests for categorical variables. For all-cause mortality or overall survival (OS), survival time was defined as a continuous variable from the date of diagnosis to the date of death due to any cause, or until the end of follow-up for those alive. For esophageal cancer-specific mortality or survival (CSS), survival time was measured from the date of diagnosis until date of death due to this cancer or related complications. Patients who died from causes other than EAC (or from unknown causes) were censored at the date of death, while those alive were censored at last clinical contact or end of follow up. Survival distributions were visualized using Kaplan–Meier curves, and log-rank tests were performed to assess heterogeneity (Figure 2 and Figure S1). Cox proportional hazards (CPH) regression models were used to estimate the associations between clinical and lifestyle exposures and OS or CSS. Hazard ratios (HRs) with corresponding 95% confidence intervals (CIs) were obtained from models adjusted for age, sex, stage, treatment, and comorbidities. These covariates were selected a priori based on prior studies [12,16,17,18,19]. When analyzing survival associations for smoking and PA, we assessed alcohol consumption and BMI as potential confounders in the respective multivariable models. Since effect estimates did not change by 10%, these variables were not retained in final models. The proportional hazards (PH) assumption was evaluated for all independent covariates using a goodness-of-fit test proposed by Schoenfeld and implemented in the R package Survival. The PH assumption was not violated when analyzing patients with localized/regional tumors (stage I–III) separately from those with advanced/metastatic disease (stage IV), as conducted in this study. All other statistical analyses were conducted using the SAS statistical analysis software (version 9.4). *p* < 0.05 was considered statistically significant.

### 2.4. Exploratory Analysis

To assess the influence of early adulthood BMI on EAC survival, we evaluated associations between OS and self-reported body weight, which was used to calculate BMI at ages 18, 30, 45, and 60 years. Since dietary exposure data were available for only a subset of DBBR participants (2003–2016), our dietary analysis was restricted to individuals with localized/regional tumors. We examined associations between OS and self-reported frequency of consumption of fruits, vegetables, and meats. Participants identified as outliers for energy consumption were excluded from the analyses (total calorie intake <500 or >3500 kcal). HRs and 95% CIs for OS were estimated in those with ‘high’ compared to ‘low’ frequency of food intake, using the median value for frequency intake as the threshold between low and high. Using assigned values of frequency intakes per month for each food, we estimated the p-for-trend values. CPH models were utilized to estimate the association between energy-adjusted macronutients (%kcal) categorized as tertiles and OS. P for trend was estimated using continuous nutrient values. All multivariable models were adjusted for age, sex, stage, treatment, comorbidities and energy intake (in kcals). 

## 3. Results

### 3.1. Study Participant Characteristics

Demographic, lifestyle, and clinical characteristics of included patients are presented in Table 1 and Table 2. Among the 371 participants, 86% were male, ~94% were White, and the median age at diagnosis was 64.9 years. In total, 58% were former smokers, and 22% were current smokers. Based on BMI measured around the time of diagnosis, 33% were overweight (BMI 25–29.99) and 43% were obese (BMI ≥ 30). A total of 30% of patients reported involvement in some form of MVPA in the decade before diagnosis (Table 1). At the end of the follow-up period, 72.2% of the patients were deceased. Of these, 77% died from this cancer or its complications (cancer-specific deaths). Additionally, 64% of the tumors were located in the lower third of the esophagus, and 31% at the GEJ/cardia. Median survival for the entire sample was 20 months. Approximately 41% of participants (*n* = 145) had advanced/metastatic disease (stage IV), while 59% had localized/regional tumors (stage I = 14.8%, stage II = 17.9%, stage III = 26.8%) (Table 2). Several variables exhibited significant differences between alive versus deceased patients based on Chi-squared/ANOVA and t-tests—stage at diagnosis, tumor location, treatment (*p* < 0.05); differences in age at diagnosis, smoking, current PA, MVPA, comorbidities satisfied *p* < 0.10 (Table S3). Median survival time in months and the corresponding 95% confidence interval for each categorical variable were computed.

### 3.2. Localized/Regional Tumors

Among individuals diagnosed with localized/regional disease (stage I, II, or III), several factors were associated with increased risk of all-cause mortality, in mutually adjusted multivariable CPH regression models—older age (>50 years), male sex, diagnosis at stage II or III, and non-receipt of surgical treatment (Table S4). In models adjusted for age, sex, stage, treatment, and comorbidities, current smokers at the time of diagnosis had a 2.5-fold increased risk of all-cause mortality, compared to never smokers (HR = 2.5 [1.42–4.53], *p* = 0.002). BMI at diagnosis, history of alcohol consumption, medication use, and pre-diagnostic MVPA were not associated with OS. Individuals who reported being currently active at the time of questionnaire completion had a 42% reduced risk of all-cause mortality compared to those who were currently inactive (HR = 0.58 [0.35–0.96], *p* = 0.035) (Table 3). Similar results were obtained in the analysis of cancer-specific mortality (Table S5).

### 3.3. Advanced/Metastatic Tumors

Among individuals diagnosed with advanced/metastatic disease (stage IV), no survival associations were observed for age, sex, treatment, or comorbidities, in multivariable CPH regression models (Table S4). In addition, no survival associations were identified for modifiable lifestyle exposures such as smoking, alcohol consumption, BMI at diagnosis, and physical activity, or for use of anti-reflux medications. Interestingly, patients reporting pre-diagnostic use of statins had a 38% reduced risk of all-cause mortality compared to statin non-users (HR = 0.62 [0.42–0.92], *p* = 0.018). Similarly, patients reporting pre-diagnostic use of NSAIDs (including aspirin) had a 39% reduced risk of all-cause mortality relative to NSAID non-users (HR = 0.61 [0.42–0.91], *p* = 0.016) (Table 3). The observed associations remained significant after including both statins and NSAIDs in the same model, and after additional adjustment for smoking status and BMI. Similar results were obtained when analyzing cancer-specific mortality (Table S5).

### 3.4. Exploratory Analyses

Consistent with findings from EMR data, self-reported BMI around diagnosis (ascertained from DBBR questionnaires) was not associated with OS. Similarly, BMI at age 18, 30, 45, or 60 years was not linked to OS (Table S6). Dietary consumption of over three servings/month of broccoli and carrots was associated with a 47% and 62% reduced risk of all-cause mortality, respectively, after adjustment for age, sex, stage, treatment, comorbidities, and energy intake (Table S7). When examining P for trend, we found that a unit increase in serving number per month of total vegetables (including potatoes), or corn, was associated with improved OS, whereas yogurt intake was associated with reduced OS. No survival associations were noted for intake of other fruits, vegetables, or meats. Individuals who consumed more than 18.6 g of fiber per day had a 66% reduced risk of all-cause mortality, but this association was not observed when fiber intake was analyzed as a percent of total kilocalories (Table S7).

## 4. Discussion

Despite diagnostic and therapeutic advances, the average 5-year survival for EAC remains poor. Given the limited and inconsistent evidence from past studies (Table S8), this study sought to investigate the influence of clinical and pre-diagnostic lifestyle exposures on EAC prognosis. We report that among patients with localized/regional tumors (stages I–III), current smoking was associated with reduced overall and cancer-specific survival, while current physical activity was linked to improved overall prognosis. By contrast, among patients with stage IV tumors, pre-diagnostic use of statins or NSAIDs was associated with increased overall and cancer-specific survival. Our exploratory analysis of dietary exposures among patients with localized/regional disease suggested a survival benefit associated with high intakes of broccoli, carrots, and fiber prior to diagnosis. Consistent with results of prior studies, stage and treatment were strong prognostic factors in our analyses, while alcohol consumption was not associated with survival [12,13,16,18,19].

A unique feature of this study was our assessment of EAC survival associations separately among patients with localized/regional versus advanced/metastatic disease, when considering smoking, physical activity, medication use, and diet. The rationale for this approach was two-fold. First, stage IV patients exhibited significant differences by treatment type and survival time, relative to other EAC patients. For example, ~66% of patients diagnosed with localized/regional EAC received surgery, as compared to only 3% of those with advanced/metastatic (stage IV) disease. Second, the stage variable did not satisfy the proportional hazards assumption when patients of all stages were pooled together as a single group. However, the PH assumption was not violated when analyzing patients with local/regional tumors (stages I–III) separately from those with stage IV cancer. Notably, our stratified approach identified certain survival associations which were only apparent among cases with localized/regional disease, and other associations only observed among patients with advanced/metastatic disease. To our knowledge, analyses of this form have not been conducted in prior studies of EAC survival.

Among lifestyle exposures, we identified a significantly increased risk of all-cause and cancer-specific mortality among current smokers with localized/regional tumors. Similar to findings reported for colorectal cancer [37], smoking was not associated with higher mortality among patients with advanced stage disease, most of whom have poor outcomes and limited survival. Smoking is a well-established EAC risk factor [38]. Results from EAC survival studies have been mixed, with some reports suggesting reduced survival among smokers, and others finding no association [12,16,18,19]. Missing data for stage has been a complicating factor in certain studies, limiting the ability to perform stratified analyses. Several studies have shown that smoking is associated with increased mortality among cancer patients, including a large study of >5000 patients, encompassing a wide range of malignancies [39].

Consistent with prior EAC-specific studies [12,18], we did not find an association between pre-diagnostic moderate-vigorous physical activity (MVPA) and overall survival. However, pre-diagnostic MVPA did correlate with improved cancer-specific survival (HR = 0.41, *p* = 0.04), among patients with localized/regional tumors. We further found that patients who reported being currently active (around the time of questionnaire completion) had improved overall survival relative to those who were currently inactive. Importantly, this association remained unchanged after adjusting for BMI and smoking status, reducing the likelihood of confounding by these factors. Similar, but borderline-significant results were obtained for cancer-specific survival (HR = 0.51, *p* =0.069). Larger sample sizes are needed to support more robust analysis of physical activity in relation to all-cause versus cancer-specific mortality. A recent study suggested that habitual PA, i.e., before and after cancer diagnosis, was associated with reduced all-cause mortality for bladder, breast, colorectal, esophageal, prostate, skin, endometrial (uterine), and ovarian cancers. This study did not investigate associations with specific histological subtypes [25]. Whether or not post-diagnostic physical activity has any influence on EAC survival has not been determined, but remains an important research question, as patients could be counseled at the time of diagnosis, or enrolled in an intervention, to increase their activity. Biological mechanisms underlying associations between PA and cancer survival are under active investigation and may include reduced chronic inflammation and improved insulin sensitivity, metabolic function, and immune surveillance [25,40,41]. Our analyses uncovered survival associations with smoking and PA among patients with localized/regional tumors, but not advanced/metastatic disease. In stage IV patients, the aggressive nature of their illness may overwhelm potential influences of these lifestyle behaviors.

This was the first EAC study to assess stage-specific survival associations with pre-diagnostic statin and NSAID use. Among stage IV patients, ever-use of either class of medication was linked to improved overall and cancer-specific survival. The protective associations remained significant after mutual adjustment, and after further adjustment for smoking status and BMI. Interestingly, no evidence of such associations was observed among patients with localized/regional tumors (adjusted hazard ratios, 1.00 and 1.12). Prior studies have indicated that use of NSAIDs or statins is associated with reduced risk of EAC [42,43,44,45]. Post-diagnostic use has also been linked to improved survival in EAC [27,28,46], but associations with pre-diagnostic use have not been reported [12,18,19].

NSAIDs including aspirin are anti-inflammatory drugs that inhibit COX enzymes, reducing cytokine/chemokine production and cell proliferation [47]. Genomic studies of patients with BE have demonstrated lower levels of somatic chromosome alterations among NSAID users versus non-users, suggesting potential beneficial effects on genome integrity [48]. Pre-diagnostic NSAID use has also been linked to lower risk of metastasis in other cancers such as breast and prostate [49]. Statins are cholesterol-lowering mediations that act through inhibition of the HMG CoA reductase enzyme. Statins exert both anti-proliferative and pro-apoptotic effects and may hinder neoplastic progression through multiple mechanisms, including increased ER stress, mTOR inhibition, caspase induction, and NF-kB blockade [43,50]. Improved survival has also been reported among statin users with stage IV lung cancer [51].

Consistent with some prior studies [12,23], we did not find an association between BMI at diagnosis and OS. Others have reported that a higher BMI at diagnosis was associated with improved survival [18,19,22]. In contrast to two earlier reports, we also found no association between self-reported past adulthood BMI and OS [17,52]. Inconsistencies may be related to the time of exposure ascertainment, exposure misclassification due to self-report, and inability to predict the precise biological window of carcinogenesis. Mechanisms underlying a potential positive or negative role for obesity in EAC prognosis remain poorly understood but may be linked to improved nutritional status or increased systemic inflammation, respectively [17].

In exploratory analyses, we found that consumption of over three servings of broccoli or carrots, and higher total dietary fiber intake, was associated with reduced risk of all-cause mortality among patients with localized/regional EAC. Similar to a small number of prior studies, we did not find evidence of survival associations with dietary macronutrients such as sugar and carbohydrates [26,53,54]. We also observed improved overall survival with increasing frequency of intake of total vegetables, and among patients with high (versus low) total dietary fiber. This latter association may be confounded by energy as it was not observed when analyzing total fiber as a percentage of kilocalories, although a borderline consistent trend was noted (*p* = 0.07). Intake of cruciferous vegetables such as broccoli has been linked to improved survival among bladder and breast cancer patients [55,56]. Isothiocyanate, the glucosinolate-derived bioactive compound present in broccoli extract, has anti-cancer properties linked to induction of phase 2 detoxification enzymes [55]. Prospective studies have also suggested reduced mortality among breast and colon cancer patients with fiber intake [57,58]. Dietary fiber may reduce pro-inflammatory cytokines in the tumor microenvironment; improve insulin sensitivity, lipid metabolism, and endothelial function; produce gastrointestinal butyrate which modulates the immune response [58]. We acknowledge that the survival associations reported for dietary exposures (e.g., broccoli, carrots, fiber) may also reflect an overall healthy lifestyle, and not the influence of specific foods per se. Dietary associations identified in our report warrant further investigation and require validation in larger patient cohorts.

Our study has several strengths. A sample size of 371 patients was sufficiently large to allow for stratified analyses to be conducted by localized/regional versus advanced/metastatic staging groups. This approach enabled us to identify survival associations which may have been obscured in past analyses and gain further insight into relevant prognostic factors in clinically distinct patient subgroups. Availability of data from both electronic medical records and epidemiologic questionnaires facilitated extensive assessment of pre-diagnostic factors such as physical activity, medications, and diet, while also providing for relatively complete information for important covariates such as stage and treatment, which had substantial missingness in previous studies. Since the majority of EAC patients died from this cancer or its complications, and our models were adjusted for comorbidities, bias due to competing risks is less likely. 

Our study also has certain limitations. Exposure ascertainment from the EMR and the DBBR questionnaire is subject to measurement error, which may reflect recall bias on the part of patients answering questions about past health history, inaccuracies in the medical record, or abstraction errors. Nevertheless, we observed a high concordance rate across duplicate abstractions of a limited sample of records and note that differential recall based on the severity of disease or length of survival is unlikely. Some level of selection bias is possible since voluntary participation in DBBR may introduce a healthy cohort effect wherein individuals with less severe disease are more inclined to participate. Results may not be generalizable since most patients were White and received treatment at a comprehensive cancer center offering state-of-the-art surgical and medical therapies. Lack of information on, or accounting for, post-diagnostic factors such as weight change, smoking cessation, physical activity, diet, and medications may have resulted in residual confounding. Consistent with prior reports (Table S8), we chose to assess raw *p* values rather than impose stringent corrections for multiple comparisons, given the high biologic plausibility of survival associations with the limited number of exposures considered in our main analysis. Our decision to jointly analyze adenocarcinomas of the esophagus and GEJ/cardia, to maximize statistical power, restricted our ability to identify survival relationships specific to one subgroup. Nevertheless, demographic and clinical characteristics, such as stage at diagnosis, treatments received, and survival time, did not differ between these groups.

## 5. Conclusions

In conclusion, this study assessed the impact of modifiable lifestyle and clinical factors on survival among EAC patients, while adjusting for key covariates. Our findings indicate that lifestyle habits such as current smoking and current physical inactivity are associated with reduced survival among patients with localized/regional disease (stages I-III). Among those with advanced tumors, prior use of statins or NSAIDs was linked to improved survival. Future studies of larger patient populations are required to validate these findings, assess the influence of other treatment modalities such as tumor ablative therapies, minimally invasive surgery, and immunotherapy, and establish an evidence base to motivate clinical trials among BE and localized/regional EAC patients. Results from this study suggest that lifestyle interventions such as smoking cessation, exercise regimens, and use of cholesterol-lowering (statin) or anti-inflammatory (NSAID) medications may improve outcomes for this deadly cancer.

## Figures and Tables

**Table 1:** EAC patient characteristics.

		*n*	Mean (SD) or %
Age at diagnosis		371	64.6 (10.4)
	<50 years	28	7.5
	50–70 years	228	61.5
	>70 years	115	31.0
Sex	Female	52	14.0
	Male	319	86.0
Race	White	349	94.1
	Black	6	1.6
	Other/not defined	16	4.3
BMI (kg/m^2^)	<24.99	89	24.4
	25–29.99	120	32.9
	>30	156	42.7
Smoking status	Never	73	20.1
	Former	212	58.4
	Current	78	21.5
Smoking pack-years	0–15	49	21.9
(ever smokers)	15–30	47	21.0
	30–45	52	23.2
	>45	76	33.9
Alcohol use	Never	61	18.5
	Social drinker	201	61.1
	Heavy drinker	67	20.4
Physical activity: Pre-diagnostic *	No	120	69.8
(moderate/vigorous)	Yes	52	30.2
Physical activity: Current *	No	88	50.9
	Yes	85	49.1
Comorbidities	None	124	33.4
	One or more	247	66.6
H/o antacid use	No	136	37.1
	Yes	231	62.9
H/o statin use	No	212	57.8
	Yes	155	42.2
H/o NSAID use	No	237	64.6
	Yes	130	35.4
Education *	High school	80	46.5
	Some college	58	33.7
	College/advanced degree	34	19.8
Family history of EC	No	352	96.2
	Yes	14	3.8
Family history: other cancers	No	125	34.2
	Yes	241	65.8

Numbers may not add to total due to missing data. * Obtained from DBBR epidemiological questionnaires. Other variables obtained from EMR (RP-BEAR survival arm).

**Table 2:** Clinical and outcome variables.

		*n*	Mean (SD) or %
Tumor location	Upper third	1	0.3
	Middle third	17	4.6
	Lower third	235	64.0
	GEJ/cardia	114	31.1
Stage at diagnosis	I	53	14.8
	II	64	17.9
	III	96	26.8
	IV	145	40.5
Grade	Well differentiated	13	3.6
	Moderately differentiated	165	45.2
	Poorly differentiated	187	51.2
Vital status **	Alive	103	27.8
	Deceased	267	72.2
Median survival (months) **		370	20 (33.1)
Cause of death **	EAC/complications	205	76.8
	Other causes	21	7.9
	Unknown	41	15.4
Endoscopic therapy (PDT/EMR/RFA)	No	317	87.6
	Yes	45	12.4
Surgical treatment	No	220	60.6
	Yes	143	39.4
Type of surgery	Standard esophagectomy	57	41.0
	Minimally invasive	82	59.0
Neoadjuvant therapy	No	14	9.9
	Yes	128	90.1
Type of neoadjuvant	Chemoradiation	123	96.1
	Chemotherapy only	5	3.9
Any chemotherapy	No	80	22.3
(excluding neo-adjuvant)	Yes	278	77.7
Any radiation	No	238	66.7
(excluding neo-adjuvant)	Yes	119	33.3

H/o: history of. Numbers may not add to total due to missing data. ** Obtained from Roswell Park tumor registry. All other variables obtained from EMR (RP-BEAR survival arm).

**Table 3:** Hazard ratios and 95% CIs for overall survival associations with modifiable lifestyle exposures.

		Localized/Regional (Stage I, II, III) (*n* = 213) Adjusted All-Cause Mortality *					Advanced/Metastatic (Stage IV) (*n* = 145) Adjusted All-Cause Mortality **				
		Deaths/ Total	HR	95% CI		*p* Value	Deaths/ Total	HR	95% CI		*p* Value
				LL	UL				LL	UL	
Smoking history	Never Former Current	20/44 72/113 37/48	Ref 1.54 **2.54**	0.93 **1.42**	2.56 **4.53**	0.097 **0.002**	24/26 80/88 22/25	Ref 1.20 0.87	0.72 0.47	1.98 1.61	0.484 0.642
Smoking pack-years (ever smokers)	<15 15-29 30-44 45+	17/26 18/25 22/31 31/42	Ref 0.82 0.89 1.02	0.39 0.53 0.53	1.61 1.99 1.87	0.524 0.927 0.980	17/20 17/18 14/19 29/30	Ref 1.86 0.99 0.82	0.90 0.45 0.41	3.83 2.16 1.65	0.094 0.972 0.579
Alcohol use	Never Social drinker Heavy drinker	26/36 71/110 23/37	Ref 0.81 0.75	0.51 0.41	1.29 1.37	0.370 0.341	23/24 73/81 23/24	Ref 1.01 0.89	0.62 0.49	1.64 1.61	0.965 0.699
BMI	<25.0 25–29.99 30+	32/45 45/66 53/96	Ref 0.84 0.79	0.53 0.50	1.34 1.24	0.465 0.305	33/37 44/49 51/55	Ref 0.76 1.34	0.47 0.86	1.21 2.10	0.242 0.198
Physical activity: Pre-diagnostic (moderate/vigorous)	No Yes	48/61 21/34	Ref 0.69	0.39	1.21	0.197	48/52 17/18	Ref 0.89	0.51	1.56	0.628
Physical activity: Current	No Yes	40/46 30/50	Ref **0.58**	**0.35**	**0.96**	**0.035**	33/37 32/33	Ref 1.17	0.70	1.96	0.550
Prior antacid use	No Yes	43/72 87/135	Ref 1.09	0.75	1.61	0.648	53/57 75/84	Ref 1.00	0.69	1.46	0.997
Prior statin use	No Yes	75/120 55/87	Ref 1.00	0.68	1.46	0.993	74/81 54/60	Ref **0.62**	**0.42**	**0.92**	**0.018**
Prior NSAID use	No Yes	90/140 40/67	Ref 1.12	0.76	1.65	0.575	80/84 48/57	Ref **0.61**	**0.42**	**0.91**	**0.016**

* Adjusted for age, sex, stage, treatment (surgery and chemotherapy [excluding neoadjuvant]), and comorbidities. ** Adjusted for age, sex, treatment (chemotherapy), and comorbidities. Ref, reference group. Statistically significant associations (*p* < 0.05) are in bold.

## Data Availability

The data presented in this study are available upon reasonable request from the corresponding author. The data are not publicly available due to privacy concerns.

## Supplementary Materials

The following are available online at https://www.mdpi.com/article/10.3390/cancers13184653/s1, Figure S1: Kaplan-Meier survival curves by exposure status, Table S1: List of variables and corresponding data sources, Table S2: Patient comorbidities by stage at diagnosis, Table S3: Patient characteristics by vital status (n=367), Table S4: Survival associations with age, sex, stage, treatment, comorbidities, Table S5: Cancer-specific survival associations with modifiable lifestyle exposures, Table S6: Survival associations with BMI at specified ages during the lifecourse, Table S7: Survival associations with dietary intake of foods, Table S8: Summary of past EAC survival studies.
