# Supplementary material for: Clinical and Lifestyle-Related Prognostic Indicators among Esophageal Adenocarcinoma Patients Receiving Treatment at a Comprehensive Cancer Center
Source: Cancers (Basel). 2021 Sep 16;13(18):4653. doi: 10.3390/cancers13184653 (PMC8465866; doi:10.3390/cancers13184653)
Supplement: Supplementary file 1 [file cancers-13-04653-s001.zip › cancers-1293800-supplementary.pdf]

SUPPLEMENTARY DATA

Figure S1. Kaplan-Meier survival curves by exposure status.

Table S1. List of variables and corresponding data sources.

Table S2. Patient comorbidities by stage at diagnosis

Table S3. Patient characteristics by vital status (n=367).

Table S4. Survival associations with age, sex, stage, treatment, comorbidities.

Table S5. Cancer-specific survival associations with modifiable lifestyle exposures.

Table S6. Survival associations with BMI at specified ages during the lifecourse.

Table S7. Survival associations with dietary intake of foods.

Table S8. Summary of past EAC survival studies.

Figure S1. Kaplan-Meier survival curves by exposure status.

A) By stage at diagnosis: all eligible patients

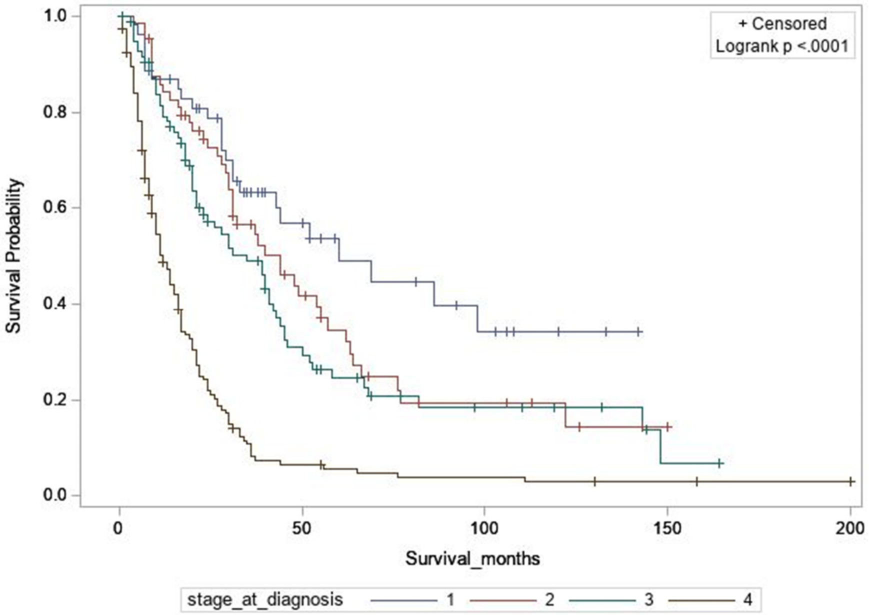

B) By pre-diagnostic statin use: localized/regional tumors

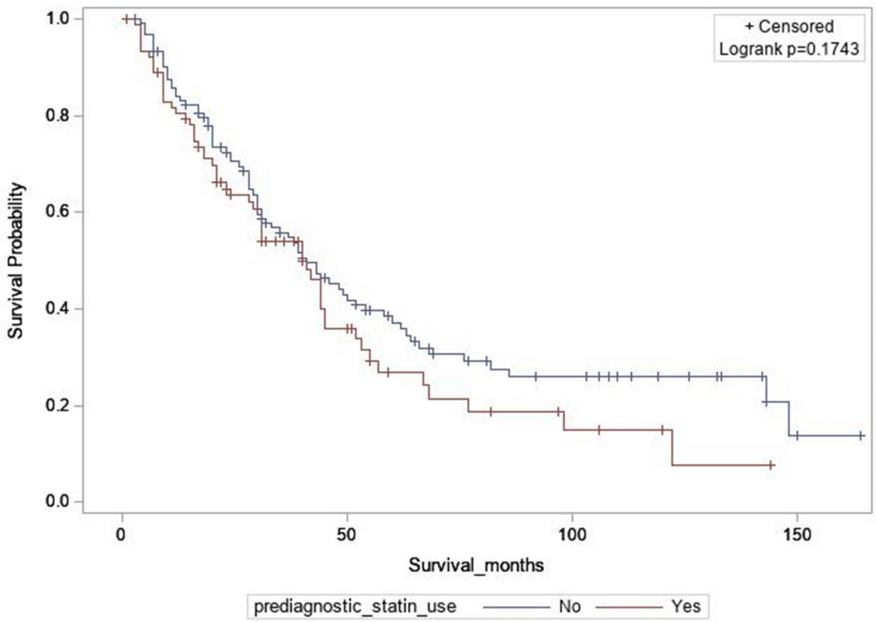

C) By pre-diagnostic statin use: advanced/metastatic tumors

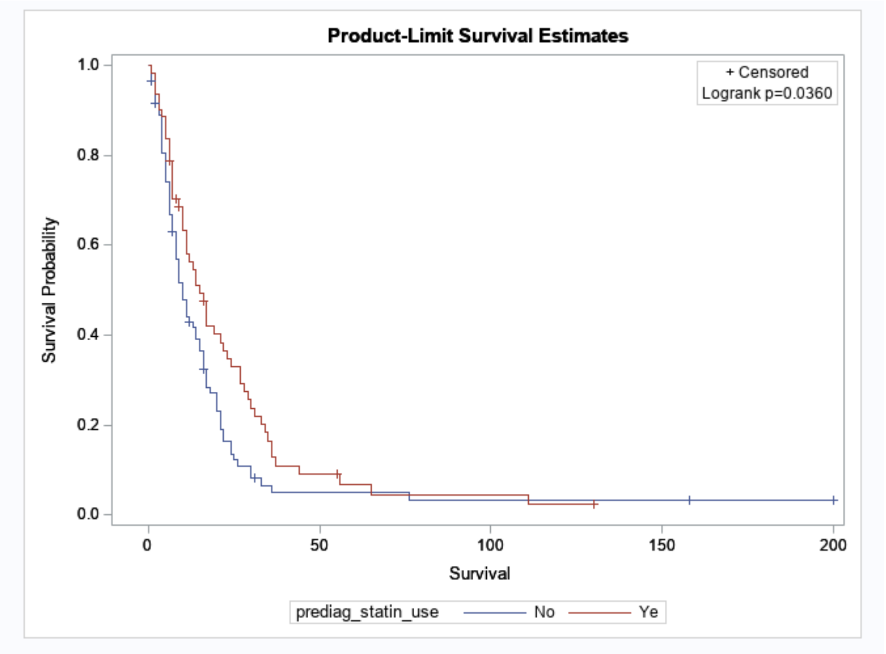

D) By pre-diagnostic NSAID use: localized/regional tumors

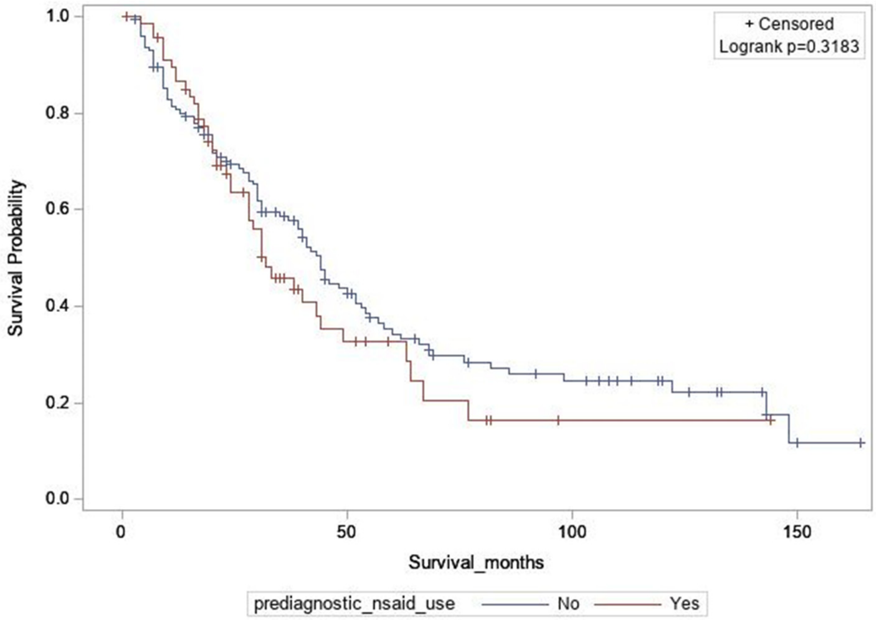

E) By pre-diagnostic NSAID use: advanced/metastatic tumors

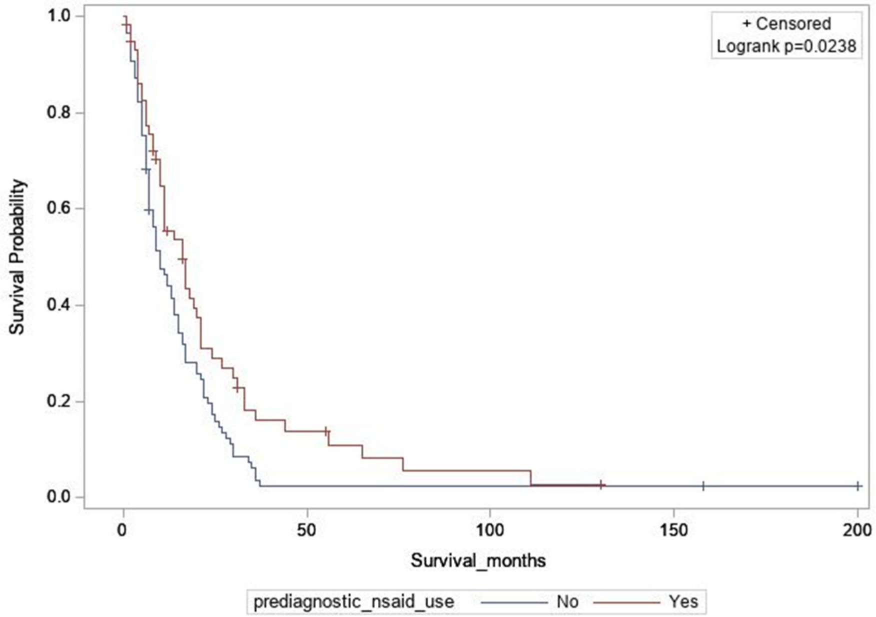

**Table S1. List of variables and corresponding data sources.**	
**EMR**	• Date of diagnosis<br>• Age at diagnosis<br>• Race<br>• Sex<br>• Tumor location<br>• Stage at diagnosis<br>• Grade at diagnosis<br>• Family history of EAC or other cancers<br>• Smoking status at time of diagnosis<br>• Smoking intensity at time of diagnosis<br>• History of alcohol consumption<br>• BMI at diagnosis<br>• Comorbidities (none vs one or more): history of cardiovascular disease, pulmonary disease, diabetes, kidney disease, liver disease and cerebrovascular disease<br><u>Pre-diagnostic Medication history</u><br>• Anti-reflux medicines: Does the patient have previous history of antacid use?<br>• NSAIDs: Does the patient have previous history of NSAID use (aspirin included)?<br>• Statins: Does the patient have previous history of statin use?<br><u>Treatment variables</u><br>• Local ablative treatments (photo-dynamic therapy [PDT], radio-frequency ablation [RFA], endoscopic mucosal resection [EMR])<br>• Neo-adjuvant therapy administered<br>• Type of neo-adjuvant therapy<br>• Surgery<br>• Type of surgery<br>• Any chemotherapy (excluding neo-adjuvant)<br>• Any radiation (excluding neo-adjuvant)
**DBBR**	• Physical activity (moderate/vigorous): Pre-diagnostic (decade prior to diagnosis)<br>• Physical activity: Current<br>• Self-reported weight at age 18yrs, 30yrs, 45 yrs, 60 yrs<br>• Dietary consumption habits in the year prior (servings/week or servings/month)
**Tumor Registry**	• Vital status: Alive vs deceased<br>• Survival time (in months)

Table S2. Patient comorbidities by stage at diagnosis			
		**Localized/regional (Stage I, II, III) (n=213)**	**Advanced/metastatic (Stage IV) (n=145)**
		n (%)	n (%)
**Cardiovascular disease**	No	126 (59.4)	97 (66.9)
	Yes	86 (40.6)	48 (33.1)
**Diabetes mellitus**	No	151 (71.2)	101 (69.7)
	Yes	61 (28.8)	44 (30.3)
**Lung disease**	No	149 (70.3)	120 (82.8)
	Yes	63 (29.7)	25 (17.2)
**Kidney disease**	No	187 (91.2)	130 (92.2)
	Yes	18 (8.8)	11 (7.8)
**Liver disease**	No	197 (95.2)	135 (93.1)
	Yes	10 (4.8)	10 (6.9)
**Cerebrovascular disease**	No	185 (88.1)	136 (93.8)
	Yes	25 (11.9)	9 (6.2)

Numbers may not add to total due to missing data.
Obtained from EMR organized under RP-BEAR survival arm.

Table S3. Patient characteristics by vital status (n=367).					
**Characteristics**		**Deceased (n=267) n (%) or mean (SD)**	**Alive (n=103) n (%) or mean (SD)**	**P value***	**Median survival (95% CI) In months**
**Age at diagnosis**		65.1 (10.6)	63.2 (10.0)	**0.098**	20 (17-22)
	< 50yrs	18 (6.7)	10 (9.7)	0.442	19 (15-55)
	50-70yrs	162 (60.7)	65 (63.1)		20 (17-24)
	Over 70yrs	87 (32.6)	28 (27.2)		17 (10-22)
**Sex**	Female	33 (12.4)	19 (18.4)	0.131	25 (17-41)
	Male	234 (87.6)	84 (81.6)		18 (16-21)
**Race**	White	255 (95.5)	94 (91.3)	0.237	20 (17-23)
	Black	4 (1.5)	2 (1.9)		16 (4-40)
	Others/not defined	8 (3.0)	7 (6.8)		7 (2-21)
**Education**	High school	70 (50.0)	10 (31.3)	0.141	27 (17-40)
	Some college	45 (32.1)	13 (40.6)		21 (15-30)
	College/advanced degree	25 (17.9)	9 (28.1)		32 (26-54)
**BMI (kg/m^2)**	<24.99	69 (25.8)	20 (20.6)	0.130	16 (10-24)
	25-29.99	92 (34.5)	27 (27.8)		22 (18-28)
	30 and above	106 (39.7)	50 (51.5)		19 (16-23)
**Smoking status**	Never	45 (17.1)	28 (28.6)	**0.051**	24 (20-34)
	Former	159 (60.2)	52 (53.1)		19 (14-22)
	Current	60 (22.7)	18 (18.4)		17 (16-24)
**Alcohol use**	Never	50 (20.2)	11 (13.6)	0.405	19 (14-26)
	Social drinker	148 (59.9)	52 (64.2)		21 (17-28)
	Heavy drinker	49 (19.8)	18 (22.2)		20 (14-24)
**Physical activity: Pre-diagnostic**	No	101 (72.7)	19 (57.6)	**0.090**	21 (16-28)
(moderate/vigorous)	Yes	38 (27.3)	14 (42.4)		35 (28-54)
**Physical activity: Current**	No	76 (54.3)	12 (36.4)	**0.064**	21 (17-30)
	Yes	64 (45.7)	21 (63.6)		30 (22-39)
**Family history of other cancers**	No	91 (34.1)	34 (34.7)	0.913	20 (16-24)
	Yes	176 (65.9)	64 (65.3)		19 (16-23)
**Median survival time**		23.1 (22.8)	47.9 (46.4)	**<0.001***	20 (17-22)
**Tumor location**	Upper third	1 (0.4)	0 (0.0)	**0.001***	4(-)
	Middle third	12 (4.6)	5 (5.0)		20 (15-82)
	Lower third	184 (70.5)	50 (50.0)		20 (16-22)
	GEJ/cardia	64 (24.5)	45 (45.0)		20 (14-28)
**Stage at diagnosis**	I	25 (9.5)	28 (29.5)	**<0.001***	34 (28-44)
	II	43 (16.4)	21 (22.1)		31 (28-44)
	III	64 (24.4)	32 (33.7)		21.5 (19-30)
	IV	130 (49.6)	14 (14.7)		11 (9-14)
**Grade at diagnosis**	Well differentiated	7 (2.6)	6 (6.1)	0.146	33 (9-86)
	Moderately differentiated	117 (44.0)	48 (49.0)		23 (20-30)
	Poorly differentiated	142 (53.4)	44 (44.9)		15 (12-18)
**Comorbidities**	None	82 (30.7)	41 (39.8)	**0.096**	20 (18-24)
	One or more	185 (69.3)	62 (60.2)		18 (15-22)
**H/o antacid use**	No	100 (37.5)	36 (36.4)	0.848	18 (14-22)
	Yes	167 (62.5)	63 (63.6)		21 (17-23)
**H/o statin use**	No	153 (57.3)	58 (58.6)	0.825	20 (16-22)

Table S3. Patient characteristics by vital status (n=367).					
**Characteristics**		**Deceased (n=267) n (%) or mean (SD)**	**Alive (n=103) n (%) or mean (SD)**	**P value***	**Median survival (95% CI) In months**
	Yes	114 (42.7)	41 (41.4)		21 (16-24)
**H/o NSAID use**	No	175 (65.5)	61 (61.6)	0.486	20 (16-24)
	Yes	92 (34.5)	38 (38.4)		19 (17-23)
**Surgical treatment**	No	188 (70.7)	31 (32.3)	**<0.001***	12 (10-16)
	Yes	78 (29.3)	65 (67.7)		34 (28-41)
**Any chemotherapy**	No	38 (14.4)	42 (44.7)	**<0.001***	22 (19-32)
(excluding neo-adjuvant)	Yes	225 (85.6)	52 (55.3)		19 (16-22)

Numbers may not add to total due to missing data
*p value<0.05 indicates statistically significant difference by vital status

Table S4. Hazard ratios and 95% CIs for survival associations with age, sex, stage, treatment, comorbidities.											
		**Localized/regional EAC Stage I, II, III (n=213) Adjusted all-cause mortality***					**Advanced/metastatic EAC Stage IV (n=145) Adjusted all-cause mortality****				
		**Deaths/ total**	**HR**	**95% CI LL**	**95% CI UL**	**P value**	**Deaths/ total**	**HR**	**95% CI LL**	**95% CI UL**	**P value**
**Age at diagnosis (years)**		132/213	1.02	1.00	1.04	0.073	130/144	1.00	0.99	1.02	0.679
**Sex**	Male	114/181	Ref				115/125	Ref			
	Female	18/32	0.47	0.27	0.80	**0.006**	15/19	0.90	0.52	1.55	0.700
**Stage**	I	25/53	Ref								
	II	43/64	2.24	1.31	3.82	**0.003**	-	-	-	-	-
	III	64/96	2.75	1.68	4.50	**<.0001**					
**Any surgery**	No	57/72	Ref				126/138				
	Yes	75/138	0.30	0.18	0.48	**<.0001**	3/5	-	-	-	-
**Neoadjuvant therapy[a]**	No	5/13	Ref				1/1				
	Yes	70/124	1.35	0.44	4.10	0.599	2/4	-	-	-	-
**Any chemotherapy**	No	33/72	Ref				5/7	Ref			
(excluding neo-adjuvant)	Yes	97/135	0.76	0.47	1.20	0.239	123/134	0.80	0.31	2.04	0.604
**Comorbidities**	None	36/64	Ref				45/49	Ref			
	One or more	94/143	1.25	0.84	1.86	0.282	83/92	0.94	0.64	1.39	0.761

*Localized/regional: Mutually adjusted for age, sex, stage, any surgery, any chemotherapy (excluding neoadjuvant), comorbidities
**Advanced/metastatic: Adjusted for age, sex, any chemotherapy (excluding neoadjuvant) and comorbidities
a: Adjusted for age, sex, stage, any chemotherapy (excluding neoadjuvant), comorbidities

Table S5. Hazard ratios and 95% CIs for cancer-specific survival associations with modifiable lifestyle exposures.												
		**Localized/regional (Stage I, II, III) (n=213) Adjusted cancer specific mortality***					**Advanced/metastatic (Stage IV) (n=145) Adjusted cancer specific mortality****					
		**Deaths/ total**	**HR**	**95% CI**		**P value**	**Deaths/ total**	**HR**	**95% CI**		**P value**
				**LL**	**UL**				**LL**	**UL**	
**Smoking history**	Never	11/44	Ref				23/26	Ref			
	Former	44/113	1.77	0.90	3.48	0.100	72/88	1.15	0.69	1.93	0.591
	Current	26/48	**3.02**	**1.43**	**6.37**	**0.004**	22/25	0.89	0.48	1.68	0.713
**Smoking pack-years**	<15	12/26	Ref				16/20	Ref			
(ever smokers)	15-29	11/25	0.88	0.37	2.07	0.763	16/18	1.74	0.83	3.67	0.144
	30-44	14/31	1.01	0.45	2.27	0.979	13/19	0.92	0.41	2.07	0.848
	45+	20/42	1.09	0.50	2.36	0.836	27/30	0.80	0.39	1.63	0.537
**Alcohol use**	Never	18/36	Ref				20/24	Ref			
	Social drinker	46/110	0.77	0.44	1.36	0.372	67/81	1.05	0.63	1.75	0.856
	Heavy drinker	12/37	0.58	0.27	1.25	0.164	23/24	1.00	0.54	1.84	0.998
**BMI**	<25.0	17/45	Ref				32/37	Ref			
	25-29.99	27/66	0.99	0.54	1.85	0.989	41/49	0.74	0.45	1.19	0.212
	30+	37/96	1.13	0.63	2.02	0.695	46/55	1.28	0.81	2.04	0.292
**Physical activity: Pre-diagnostic**	No	28/61	Ref				44/52	Ref			
(moderate/vigorous)	Yes	9/34	**0.41**	**0.18**	**0.96**	**0.041**	15/18	0.8	0.48	1.57	0.634
**Physical activity: Current**	No	22/46	Ref				30/37	Ref			
	Yes	15/50	0.51	0.24	1.06	0.069	29/32	1.17	0.68	2.01	0.566
**Prior anti-reflux Rx use**	No	26/72	Ref				50/57	Ref			
	Yes	55/135	1.25	0.77	2.02	0.376	69/84	1.00	0.68	1.48	0.994
**Prior statin use**	No	50/120	Ref				72/81	Ref			
	Yes	31/87	0.90	0.55	1.47	0.664	47/60	**0.57**	**0.38**	**0.85**	**0.007**
**Prior NSAID use**	No	55/140	Ref				77/84	Ref			
	Yes	26/67	1.16	0.71	1.90	0.549	42/57	**0.58**	**0.38**	**0.88**	**0.010**

*Adjusted for age, sex, stage, treatment (surgery and chemotherapy [excluding neoadjuvant]), and comorbidities
**Adjusted for age, sex, treatment (chemotherapy), and comorbidities

Table S6. Hazard ratios and 95% CIs for survival associations with BMI at specified ages during the lifecourse.											
**Parameters**		**Localized/regional EAC Stage I +stage II +stage III (n=213) Adjusted all-cause mortality***					**Advanced/metastatic EAC Stage IV (n=145) Adjusted all-cause mortality****				
		**Deaths/ total**	**HR**	**95% CI**		**P value**	**Deaths/ total**	**HR**	**95% CI**		**P value**
				**LL**	**UL**				**LL**	**UL**	
BMI at diagnosis (EMR)	Normal	32/45	Ref				33/37	Ref			
	Overweight/obese	98/162	0.81	0.54	1.23	0.321	95/104	1.00	0.67	1.50	0.997
Self-reported BMI at diagnosis	Normal	21/28	Ref				17/17				
	Overweight/obese	45/64	0.95	0.55	1.64	0.840	45/50	1.02	0.57	1.83	0.947
Self-reported BMI at 18 yrs	Normal	39/64	Ref				34/36				
	Overweight/obese	15/24	1.07	0.54	2.16	0.800	11/14	0.74	0.35	1.55	0.423
Self-reported BMI at 30 yrs	Normal	30/41	Ref				24/27				
	Overweight/obese	22/33	1.16	0.60	2.23	0.663	20/22	1.59	0.79	3.19	0.192
Self-reported BMI at 45 yrs	Normal	17/24	Ref				14/16				
	Overweight/obese	41/55	1.27	0.66	2.44	0.484	31/33	1.17	0.56	2.45	0.682
Self-reported BMI at 60 yrs	Normal	13/16	Ref				7/7				
	Overweight/obese	36/47	1.04	0.49	2.19	0.921	34/35	0.90	0.36	2.25	0.815

*Adjusted for age, sex, stage, treatment (any surgery and chemotherapy [excluding neo-adjuvant]), comorbidities
**Adjusted for age, sex, treatment (chemotherapy), comorbidities

Table S7. Hazard ratios and 95% CIs for survival associations with dietary intake of foods.						
		Localized/regional (Stage I, II, III) (n=213) Adjusted all-cause mortality#				
Food	Servings per month	Events/ total	HR[a]	95% CI		P-trend
**Broccoli**	Low intake (never to 3)<br>High intake (>3)	47/61<br>19/31	Ref<br>**0.53**	**0.30**	**0.96**	0.156
**Other cruciferous vegetables**[a]	Low intake (never to 1)<br>High intake (>1)	33/43<br>33/49	Ref<br>0.83	0.49	1.43	0.156
**Red and green peppers**	Low intake (never to 1)<br>High intake (>1)	34/45<br>32/47	Ref<br>1.03	0.60	1.76	0.500
**Green or string beans**	Low intake (never to 3)<br>High intake (>3)	45/58<br>21/34	Ref<br>0.70	0.39	1.26	0.329
**Green salad**[b]	Low intake (never to 4)<br>High intake (>4)	40/51<br>27/42	Ref<br>0.82	0.50	1.37	0.161
**Carrots**	Low intake (never to 3)<br>High intake (>3)	40/55<br>26/37	Ref<br>**0.38**	**0.19**	**0.72**	0.074
**Fresh tomatoes**	Low intake (never to 4)<br>High intake (>4)	35/51<br>31/41	Ref<br>1.10	0.66	1.83	0.433
**Potatoes**	Low intake (never to 4)<br>High intake (>4)	31/44<br>36/49	Ref<br>0.91	0.53	1.57	0.482
**Green peas**	Low intake (never to 1)<br>High intake (>1)	28/42<br>37/49	Ref<br>1.03	0.59	1.79	0.423
**Corn**	Low intake (never to 3)<br>High intake (>3)	45/58<br>20/33	Ref<br>0.78	0.42	1.42	**0.048**
**Total vegetables** (with potatoes)	Low intake (never to 39)<br>High intake (>39)	41/55<br>30/42	Ref<br>0.91	0.54	1.55	**0.049**
**Total vegetables** (no potatoes)	Low intake (never to 31)<br>High intake (>31)	41/53<br>30/44	Ref<br>0.77	0.45	1.32	0.059
**Poultry**[c]	Low intake (never to 3)<br>High intake (>3)	39/51<br>28/42	Ref<br>0.99	0.54	1.83	0.962
**Eggs**	Low intake (never to 4)<br>High intake (>4)	33/47<br>35/46	Ref<br>1.55	0.90	2.64	0.120
**Beef/pork/ham/lamb**	Low intake (never to 4)<br>High intake (>4)	34/45<br>33/48	Ref<br>1.11	0.61	1.93	0.069
**White fish**[d]	Low intake (never to < 1)<br>High intake (≥1)	35/49<br>31/43	Ref<br>1.03	0.61	1.74	0.987
**Dark fish**[e]	Low intake (never to < 1)<br>High intake (≥1)	41/55<br>24/36	Ref<br>0.87	0.51	1.46	0.358

**Rice/noodles** (grains)	Low intake (never to 1)<br>High intake (>1)	27/38<br>38/53	Ref<br>1.22	0.72	2.09	0.932
**Garlic**	Low intake (never to < 1)<br>High intake (≥1)	33/39<br>32/52	Ref<br>0.69	0.40	1.19	0.269
**Butter, margarine**	Low intake (never to 4)<br>High intake (>4)	32/43<br>35/50	Ref<br>1.09	0.65	1.84	0.616
**Yogurt**	Low intake (never to 1)<br>High intake (>1)	33/46<br>34/47	Ref<br>1.58	0.93	2.67	**0.003**
**Apples**	Low intake (never to 3)<br>High intake (>3)	37/53<br>30/40	Ref<br>0.87	0.52	1.46	0.145
**Bananas**	Low intake (never to 3)<br>High intake (>3)	34/46<br>32/46	Ref<br>0.82	0.46	1.48	0.249
**Peaches**	Low intake (never to 1)<br>High intake (>1)	34/49<br>32/43	Ref<br>1.12	0.65	1.93	0.918
**Oranges**	Low intake (never to 1)<br>High intake (>1)	36/50<br>28/40	Ref<br>0.64	0.38	1.10	0.768
**Berries**	Low intake (never to 3)<br>High intake (>3)	43/60<br>23/32	Ref<br>0.94	0.52	1.68	0.641
**Other fruits**	Low intake (never to 3)<br>High intake (>3)	44/61<br>20/31	Ref<br>0.58	0.33	1.03	0.527
<u>**Total fruits**</u>	Low intake (never to 31)<br>High intake (>31)	39/52<br>32/45	Ref<br>0.88	0.51	1.54	0.374
**Milk**	Low intake (never to 4)<br>High intake (>4)	39/50<br>28/43	Ref<br>0.85	0.50	1.43	0.137
**Coffee**	Low intake (never to 1)<br>High intake (>1)	28/44<br>40/50	Ref<br>1.16	0.69	1.97	0.927
**Diet soda**	Low intake (never)<br>High intake (≥1)	42/56<br>24/36	Ref<br>0.77	0.44	1.35	0.174
**Regular soda**	Low intake (never)<br>High intake (≥1)	28/40<br>39/53	Ref<br>1.07	Ref<br>0.64	1.81	0.366
**Macronutrient (per day)**	**Tertiles of intake**	**Events**	**HR**	**LL**	**UL**	**P-trend**
**Energy intake (kcal)***	Tertile 1 (less than 1451)<br>Tertile 2 (1451-2127.9)<br>Tertile 3 (above 2127.9)	22/30<br>25/34<br>23/32	Ref<br>1.10<br>1.15	 0.57<br>0.58	 2.10<br>2.28	0.815
**Carbohydrate (% kcal)**	Tertile 1 (0-44.35)<br>Tertile 2 (44.35-51.12)<br>Tertile 3 (above 51.12)	21/31<br>26/33<br>23/32	Ref<br>1.03<br>0.81	 0.55<br>0.42	 1.93<br>1.54	0.091
**Total fiber (grams)**	Tertile 1 (0-11.50)<br>Tertile 2 (11.50-18.64)<br>Tertile 3 (above 18.64)	31/39<br>22/28<br>18/30	Ref<br>0.53<br>**0.34**	 0.27<br>**0.12**	 1.02<br>**0.93**	0.144

**Total fiber (%kcal)**	Tertile 1 (0-7.11)<br>Tertile 2 (7.11-9.06)<br>Tertile 3 (above 9.06)	25/31<br>23/33<br>22/33	Ref<br>0.89<br>0.91	 0.49<br>0.47	 1.61<br>1.73	0.070
**Total sugars (%kcal)**	Tertile 1 (0-34.86)<br>Tertile 2 (34.86-52.89)<br>Tertile 3 (above 52.89)	21/31<br>26/33<br>23/32	Ref<br>1.35<br>0.86	 0.74<br>0.47	 2.46<br>1.59	0.914
**Proteins (%kcal)**	Tertile 1 (0-13.87)<br>Tertile 2 (13.87-16.89)<br>Tertile 3 (above 16.89)	25/32<br>20/32<br>25/32	Ref<br>0.81<br>1.73	 0.43<br>0.92	 1.53<br>3.29	0.491
**Total fats (%kcal)**	(Tertile 1 (0-31.09)<br>Tertile 2 (31.09-37.82)<br>Tertile 3 (above 37.82)	21/30<br>25/34<br>24/32	Ref<br>1.30<br>1.04	 0.70<br>0.55	 2.42<br>1.96	0.898
**Saturated fatty acids (%kcal)**	Tertile 1 (0-9.93)<br>Tertile 2 (9.93-13.67)<br>Tertile 3 (above 13.67)	22/31<br>23/33<br>25/32	Ref<br>0.67<br>1.42	 0.36<br>0.75	 1.23<br>2.67	0.242
**Mono-unsaturated fatty acids (%kcal)**	Tertile 1 (0-10.88)<br>Tertile 2 (10.88-12.67)<br>Tertile 3 (above 12.67)	22/32<br>23/31<br>25/33	Ref<br>0.91<br>0.98	 0.49<br>0.53	 1.67<br>1.79	0.584
**Poly-unsaturated fatty acids (%kcal)**	Tertile 1 (0-6.17)<br>Tertile 2 (6.17-8.30)<br>Tertile 3 (above 8.30)	23/31<br>24/32<br>23/33	Ref<br>1.06<br>0.75	 0.54<br>0.40	 2.07<br>1.41	0.247

#: Adjusted for age, sex, stage, treatment (surgery and chemotherapy [excluding neo-adjuvant]), comorbidities, total energy intake
* Adjusted for age, sex, stage, treatment (surgery and chemotherapy [excluding neo-adjuvant]), comorbidities
a: Other cruciferous vegetables: cauliflower, cabbage, brussels sprouts
b: Green salad: lettuce, spinach
c: Poultry: chicken, turkey
d: White fish: halibut, cod, sole
e: Dark fish: tuna, salmon

Table S8. Summary of past studies analyzing lifestyle exposures and medication use in relation to all-cause mortality among EAC patients.					
	**Trivers et al., 2005[19]**	**Sundelof et al., 2008[18]**	**Thrift et al., 2012[12]**	**Spreafico et al., 2017[17]**	**McCain et al., 2020[16]**
**Obesity**	Pre-diagnostic BMI=25-29.9 associated with increased survival vs. BMI<25 **[HR=0.67 (0.51-0.88)]**	BMI 20 years before Dx: BMI>30 associated with increased survival vs. BMI 22-24.9 **[HR=0.60 (0.3-1.0)]**	No association*	BMI 1 year before Dx: No association*<br>Early adulthood BMI: BMI 25-30 & BMI>30 associated with reduced survival vs. BMI 18.5-25 **[HR=1.84 (1.37-2.48)]** **[HR=2.78 (1.94-3.99)]**	
**Smoking**	No association*	No association*	GE-junctional adeno: Current smoking associated with reduced survival **[HR=1.45 (1.02-2.06)]**<br>Esophageal adeno: No association*	Each 20 pack-year increase in smoking exposure associated with reduced survival **[HR=1.22 (1.15-1.43)]**	No association*
Alcohol intake	No association*	No association*	No association*		No association* [adjusted models]
Physical activity		No association*	No association*		
GERD/reflux	No association*	No association*	No association*		
NSAIDs	No association*		No association*		
Statins					

*P<0.05
